# In Vitro Non-Genomic Effects of Calcifediol on Human Preosteoblastic Cells

**DOI:** 10.3390/nu13124227

**Published:** 2021-11-25

**Authors:** Simone Donati, Gaia Palmini, Cecilia Romagnoli, Cinzia Aurilia, Francesca Miglietta, Irene Falsetti, Francesca Marini, Roberto Zonefrati, Gianna Galli, Gemma Marcucci, Teresa Iantomasi, Maria Luisa Brandi

**Affiliations:** 1 Department of Experimental and Clinical Biomedical Sciences, University of Florence, Viale Pieraccini 6, 50139 Florence, Italy; simone.donati@unifi.it (S.D.); gaia.palmini@unifi.it (G.P.); cecilia.romagnoli@unifi.it (C.R.); cinzia.aurilia@unifi.it (C.A.); francesca.miglietta@unifi.it (F.M.); irene.falsetti@unifi.it (I.F.); gianna.galli@unifi.it (G.G.); teresa.iantomasi@unifi.it (T.I.); 2 Fondazione Italiana Ricerca Sulle Malattie dell’Osso (FIRMO Onlus), 50141 Florence, Italy; francesca.marini@unifi.it (F.M.); roberto.zonefrati@unifi.it (R.Z.); 3 Bone Metabolic Diseases Unit, Department of Experimental and Clinical Biomedical, Sciences, University of Florence, AOU Careggi, 50139 Florence, Italy; gemma.marcucci@unifi.it

**Keywords:** vitamin D_3_, calcitriol, calcifediol, non-genomic effects, intracellular Ca^2+^

## Abstract

Several recent studies have demonstrated that the direct precursor of vitamin D_3_, the calcifediol [25(OH)D_3_], through the binding to the nuclear vitamin D receptor (VDR), is able to regulate the expression of many genes involved in several cellular processes. Considering that itself may function as a VDR ligand, although with a lower affinity, respect than the active form of vitamin D, we have assumed that 25(OH)D_3_ by binding the VDR could have a vitamin’s D_3_ activity such as activating non-genomic pathways, and in particular we selected mesenchymal stem cells derived from human adipose tissue (hADMSCs) for the in vitro assessment of the intracellular Ca^2+^ mobilization in response to 25(OH)D_3_. Our result reveals the ability of 25(OH)D_3_ to activate rapid, non-genomic pathways, such as an increase of intracellular Ca^2+^ levels, similar to what observed with the biologically active form of vitamin D_3_. hADMSCs loaded with Fluo-4 AM exhibited a rapid and sustained increase in intracellular Ca^2+^ concentration as a result of exposure to 10^−5^ M of 25(OH)D_3_. In this work, we show for the first time the in vitro ability of 25(OH)D_3_ to induce a rapid increase of intracellular Ca^2+^ levels in hADMSCs. These findings represent an important step to better understand the non-genomic effects of vitamin D_3_ and its role in endocrine system.

## 1. Introduction

Calcitriol (1α,25-(OH)_2_D_3_), the biologically active form of vitamin D_3_, is a hormone that participates in many biological processes, including the regulation of the serum calcium and phosphate levels, in addition to exerting direct effects on bone and mineral metabolism [1]. In bone, its actions are mediated via the interaction with the nuclear vitamin D receptor (VDR), to promote the expression of genes related to bone remodelling, such as alkaline phosphatase (ALP), type I collagen, and non-collagenous proteins [2,3,4].

VDR acts like transcription factor forming heterodimers with retinoid X receptor (RXR) and either positively or negatively regulating the expression of target genes by binding to their promoter regions through vitamin D_3_ response elements (VDREs) [2]. 

Moreover, in addition to genomic actions, all the steroid molecules have been proven to transmit via specific membrane receptors rapid non-genomic effects [5]. In 1942, Hans Selye observed that progesterone was able to induce anaesthetic effects within minutes of its administration differently than its major hormonal activity that occurred only hours after its application, thus ascertaining for the first time the non-genomic actions of steroids molecules [6]. In 1964, Spach and Streeten demonstrated that the variation of Na^+^ ions induced by aldosterone administration in dog erythrocytes happened within few minutes, offering new evidence on non-genomic actions of this steroid hormone [7]. The results of later studies remained obscure until the recent recognition of rapid non-genomic effects for various steroid hormones, including 1α,25-(OH)_2_D_3_ [8]. These actions take place on a range of seconds to minutes, are not controlled by molecules that inhibit the genomic effects (i.e., cycloheximide or actinomycin D), and occur in response even to steroids bound to large proteins and therefore no capable of entering in the cells [9].

Among the rapid non-genomic actions of 1α,25-(OH)_2_D_3_, it has been reported the stimulation of specific intracellular signal transduction pathways (i.e., mitogen-activated protein kinase (MAPK) cascades, cAMP-protein kinase A), the increase of cytoplasmic calcium concentrations (Ca^2+^), and the activation of the chloride and calcium channels [10]. 

It has been postulated that non-genomic mechanisms require the interaction between 1α,25-(OH)_2_D_3_ and intracellular or membrane-bound proteins, thereby providing efficient and rapid response to the stimulus [11].

**Figure 1:**
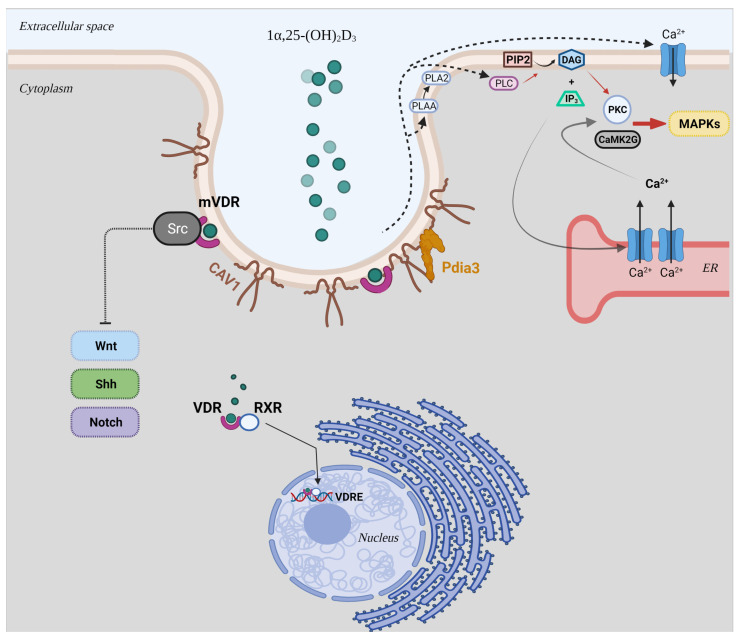
Schematic representation of the genomic and non-genomic mechanisms of the biological active form of vitamin D_3_, 1α,25-(OH)_2_D_3_. Abbreviations: mVDR: membrane-bound VDR; RXR: retinoid X receptor; VDRE: vitamin D_3_ response elements; CAV1: caveolin 1; Shh: Sonic hedgehog; Pdia3: protein disulphide isomerase family A member 3; PLA2: phospholipase A2; PLAA: PLA2 activating protein; PLC: phospholipase C; PIP2: phosphatidylinositol bisphosphate; DAG: diacylglycerol; IP_3_: inositol trisphosphate; PKC: protein kinase C; CaMK2G: calcium/calmodulin-dependent protein kinase II gamma; MAPK: mitogen-activated protein kinase.

Membrane-bound VDR (mVDR) complexed with caveolin 1 (CAV1) and proto-oncogene, non-receptor tyrosine kinase Src in caveolae is involved in the regulation of different signal transduction pathways, such as transcriptional activity of Wnt [12,13,14,15], Notch [16,17,18], and sonic hedgehog (Shh) (Figure 1) [19,20,21,22,23,24]. Moreover, its interaction with CAV1 results in the activation of several intracellular signalling molecules (i.e., phospholipase A2 (PLA2), protein kinase C (PKC), phosphatidylinositol 3-kinases (PI3Ks), calcium/calmodulin-dependent protein kinase II gamma (CaMKII), and MAP kinases) (Figure 1) [10].

In addition, these actions could be mediated via the joint interaction of CAV1 and protein disulphide isomerase family A member 3 (Pdia3) (Figure 1) [11,25,26]. This latter is an endoplasmic reticulum (ER) chaperone for calnexin (CANX) and calreticulin (CALR) and a 1α,25-(OH)_2_D_3_-membrane associated, rapid response steroid (MAARS) binding protein that interacts with vitamin D_3_ compounds, making it essential for the non-genomic responses of 1α,25-(OH)_2_D_3_. Although no binding site for 1α,25-(OH)_2_D_3_ has been identified in the structure of Pdia3 using crystallographic studies, in vivo studies on Pdia3^-/-^ and VDR^-/-^ mice strongly suggest the involvement of this ER chaperone in skeletal development and intestinal Ca^2+^ absorption [27,28,29,30,31]. On the basis of this evidence, despite the fact that Pdia3 could not directly interact with 1α,25-(OH)_2_D_3_, this protein could serve as a chaperone for vitamin D_3_ binding protein (DBP) or VDR, suggesting its importance in rapid responses to the biologically active metabolite of vitamin D_3_.

The most noticeable non-genomic rapid effect of 1α,25-(OH)_2_D_3_ is the increase of intracellular Ca^2+^ ion concentrations at subnanomolar concentrations by modulating its release from intracellular stores and its uptake in intestinal epithelia, referred as “transcaltachia” [8,32]. In addition, a rapid increase in tissue Ca^2+^ concentrations has been also found in primary cultured myocytes obtained from chicken embryonic heart [33] and in the osteoblast-like cells ROS 24/1 lacking the nuclear VDR [34].

Interestingly, evidence has demonstrated that 1α,25-(OH)_2_D_3_ exerts rapid, non-genomic actions in vivo. In particular, functional studies showed that the stimulation of phosphate uptake into intestinal epithelial cells isolated from chicken results from the rapid actions of the secosteroid hormone [35]. In a further study, Boyan et al. observed that the regulation of PKC activity from 1α,25-(OH)_2_D_3_ is mediated by rapid membrane associated mechanisms in cultured costochondral cartilage cells derived from VDR knockout mice [27].

Calcifediol (25(OH)D_3_), the major circulating form of vitamin D_3_ and the direct precursor of 1α,25-(OH)_2_D_3_, is produced mainly in the liver by hydroxylation of vitamin D_3_ through the enzyme 25-hydroxylase [36,37,38,39,40]. Recently, several studies demonstrated that 25(OH)D_3_ is an agonistic VDR ligand with gene regulatory and anti-proliferative properties, although with a lower affinity compared with the active form of vitamin D_3_ [41].

In this study we have hypothesized for the first time that the 25(OH)D_3_ could activate non-genomic pathways. As it had been previously shown, human mesenchymal stem cells (hMSCs) are an excellent model for studying the hormonal effects of 1α,25-(OH)_2_D_3_ [42], we selected MSCs derived from human adipose tissue (hADMSCs) for the in vitro analysis of the intracellular Ca^2+^ mobilization in response to 25(OH)D_3_.

## 2. Materials and Methods

### 2.1. Isolation of hADMSCs Cells and Cultures

hADMSC lines were prepared from small fragments of subcutaneous adipose tissue biopsies developed by Dr. Brandi during her visiting years at the National Institutes of Health (NIH, Bethesda, MD, USA). The adipose tissue biopsies were immediately placed in culture medium supplemented with 100 IU/mL penicillin and 100 µg/mL streptomycin and transported to the laboratory for their processing. Briefly, each sample was minced mechanically into small fragments (1 mm) and was subjected to the enzymatic treatment in Ham’s F12 Coon’s modification medium supplemented with 20% foetal bovine serum (FBS), 100 IU/mL penicillin, 100 µg/mL streptomycin, and 3 mg/mL collagenase type II for 3 h at 37 °C. After centrifugation, pellet fragments were mechanically dispersed by pipetting and sedimented by centrifugation at 400× *g* for 5 min. After removing the supernatant by aspiration, the cell pellet was suspended and cultured into a 100 mm Petri dish at 37 °C in humified atmosphere with 5% CO_2_ in growth medium (GM) [Ham’s F12 Coon’s modification medium supplemented with 10% FBS, 100 IU/mL penicillin, and 100 µg/mL streptomycin]. The medium was replaced with fresh GM every 3 days and once confluence was reached, the cells were detached by trypsinization and were used either for cell expansion, or cryopreserved upon reaching 5 × 10^3^ cells/cm^2^, or plated on tissue culture dishes or wells for other purposes.

### 2.2. Multiple Differentiation Potential Assessment of hADMSCs 

The characterization analysis to evaluate the stem cell potential of hADMSCs cell lines were performed by studying the ability of the cells to differentiate toward both the adipogenic and osteogenic lineage, as described below.

#### 2.2.1. Adipogenic Differentiation

hADMSCs cell lines were plated on 24-well plates at a cell density of 1 × 10^4^ cells/cm^2^ in GM until reaching 80–90% confluence. Afterward, the medium was changed to adipogenic medium (AM), composed as follow: Ham’s F12 Coon’s modification medium supplemented with 10%FBS, 100 IU/mL penicillin, 100 µg/mL streptomycin, 1 µM dexamethasone, 1 µM bovine insulin, 0.5 mM IsoButylMethylXanthine (IBMX), and 100 µM indomethacin. The AM was refreshed twice a week. The expression of the adipogenic phenotype was assessed on cells cultured in AM or GM (negative control) for 21 days, stained directly with freshly prepared Oil Red O working solution in order to prevent the burst of lipid droplets, and immediately observed in brightfield microscopy (Axiovert 200, Zeiss, Oberkochen, Germany). 

#### 2.2.2. Osteogenic Differentiation

hADMSCs cell lines were seeded on 24-well plates at a cell density of 1 × 10^4^ cells/cm^2^ in GM until reaching the 70–80% confluence. Afterward, the medium was switched to osteogenic medium (OM), composed as follow: Ham’s F12 Coon’s modification medium supplemented with 10% FBS (South America origin), 100 IU/mL penicillin, 100 µg/mL streptomycin, 10 nM dexamethasone, 0.2 mM sodium L-ascorbyl-2-phosphate, 10 mM β-glycerol phosphate, and 1 µg/mL calcein. The OM was refreshed twice a week. The osteogenic phenotype was evaluated up to 35 days of osteogenic induction both by monitoring alkaline phosphatase (ALP) activity and Ca^2+^ deposition by cythochemical staining. Thus, the cells were washed twice with Dulbecco’s phosphate buffered saline (DPBS), fixed in 4% paraformaldehyde (PFA)/DPBS, and washed three times with ultrapure distilled H2O. For ALP cytochemical staining, the fixed cells were washed twice with DPBS, and stained with a specific dye mixture composed of 5 mg naphtol-AS-MX phosphate sodium salt in 1mL dimethyl sulfoxide (DMSO), 40 mg Fast Red Violet LB salt in 50 mL Tris-HCl Buffer pH 9 for 30 min at 37 °C, by monitoring the course of staining every 10 min through the microscope. ALP-positive cells were stained in red, and nuclei, counterstained in green with methyl green were observed in LSCM (Zeiss, Oberkochen, Germany) under brightfield observation. For calcium mineralized deposits, hydroxyapatite (HA) deposits were stained with 1% silver nitrate solution and placed under ultraviolet light for 4 h. After that, the unreacted silver solution was removed with 5% sodium thiosulfate for 5 min and rinsed several times with distilled water. ALP+ cells and HA deposits were observed in bright field microscopy (Axiovert 200, ZEISS).

### 2.3. Evaluation of the Intracellular [Ca^2+^] Levels Variation on hADMSCs by LSCM

The variation of intracellular Ca^2+^ levels on hADMSCs exposed to 10^−5^ M of 25(OH)D_3_ was evaluated by LSCM. Briefly, cells were plated on 24-well plates at a cell density of 1 × 10^4^ cells/cm^2^ in GM, until they reaching 70% confluence on the day of imaging and were loaded with 4 × 10^−4^ M Fluo-4 acetoxymethil ester form (Fluo-4 AM) fluorescent dye (λex/λem: 494/506 nm) diluted 1/200 in Ca^2+^ free Hank’s buffered salt solution (HBSS) for 60 min at RT. Fluo-4 AM is a calcium indicator that once inside the cell, is cleaved by cellular esterases, resulting in a fluorescent form which exhibits increased fluorescence intensity at the emission wavelength of 506 nm, upon excitation at 494 nm, reflecting the cytoplasmic [Ca^2+^]. After this period, cells were washed two times with HBSS, and further incubated with 300 µL of HBSS for 60 min at RT, so that esterase activity were inhibited and allowing Fluo-4 to bind intracellular Ca^2+^. The mobilisation of intracellular Ca^2+^ was measured in untreated cells (negative control), cells exposed to 10^−5^ M of 25(OH)D_3_, and 10^−5^ M calcium ionophore-treated cells (positive control) by LSCM. Briefly, we set the LSCM to scan images every 8 s for around 8 min to establish baseline fluorescence for Fluo-4 (negative control), by exciting cells at 488 nm. Subsequently, cells were exposed to 10^−5^ M 25(OH)D_3_ added directly to a single well after 35 s of acquisition with a pipette. Once imaging was completed, the maximum intensities for Fluo-4 signals in single cells were determined by using image analysis software through the selection of multiple regions of interest (ROI). The fluorescence was then normalised by pixel-pixel adjustment to the fluorescence measured in a single image acquired before addition of the 25(OH)D_3_ (baseline fluorescence). Maximum fluorescence intensity derived from cells exposed to 25(OH)D_3_ was compared to negative control. Cells treated with 10^−5^ M calcium ionophore A23187 (Merck KGaA, Darmstadt, Germany) were used as positive control for influx of intracellular Ca^2+^ experiment.

### 2.4. Statistical Analysis

The statistical analysis was carried out by using GraphPad Prism 9 (GraphPad Software, San Diego, California, CA, USA). The normality distribution of the data was analyzed by the Kolmogorov-Smirnov and Shapiro-Wilk tests. Statistical analysis was performed by ANOVA followed by Bonferroni’s test with a predetermined experimentwise αT = 0.05.

## 3. Results

### 3.1. Isolation of hADMSCs

**Figure 2:**
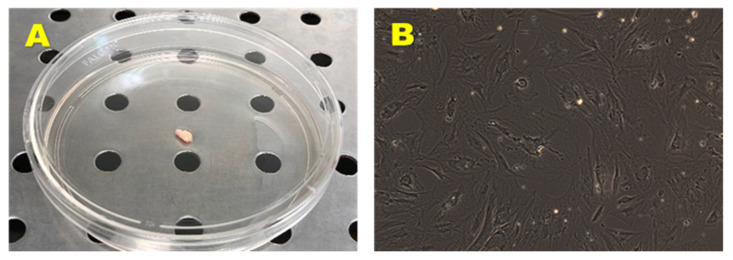
Biopsy sample obtained by surgical resection from healthy donor (**A**) and primary hADMSCs cell line (**B**). Observation with a phase contrast microscopy (AxioVision, ZEISS). Original Magnification: 10×.

From the adipose tissue biopsies (Figure 2A) have been established four cell lines, named respectively preadipocyte cell lines (PA) 60, PA 67, PA 70, and PA 73. The above cell lines displayed a spindle-shaped fibroblast-like morphology with long cytoplasmic extensions (Figure 2B). All cell lines adhered to the plastic of culture dishes without any additional surface modifications and maintained their morphology throughout their expansion.

### 3.2. Multipotentiality of hADMSCs

To evaluate the potential for their multipotentiality, cells were induced to differentiate toward the osteogenic and adipogenic phenotypes, by using appropriate medium defined in the ‘Materials and Methods’ section. 

#### 3.2.1. Osteogenic Differentiation

**Figure 3:**
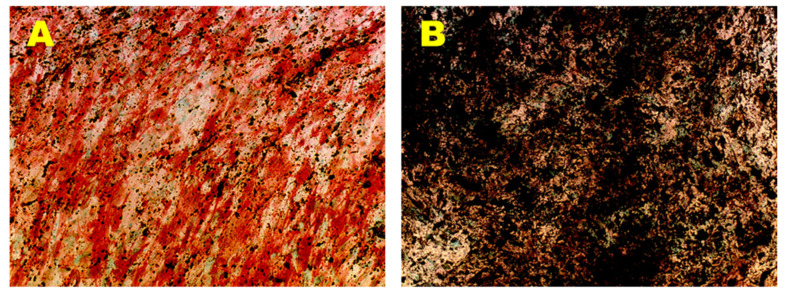
Osteogenic Differentiation Assay—ALP and HA. Osteogenic differentiation at 14 days (**A**) and 35 days (**B**) of induction by cytochemical staining for ALP with Fast Red Violet B and for HA with Von Kossa staining. The ALP+ cells are in red and the grainy deposits are in black. Nuclei are counterstained in green. Observation in brightfield (AxioVision, ZEISS). Original magnification: 20×.

Osteogenic phenotype of the established hADMSCs lines was evaluated in OM up to 35 days, by monitoring the ALP expression and the production of calcium mineralized deposits. Results obtained by cytochemical staining showed that the above-mentioned cell lines cultured in OM displayed an increase of ALP-positive cells in a time-dependent manner, reaching a maximum ALP activity at 14 days of induction (Figure 3A). In contrast, control cells cultured in GM did not exhibit any ALP-positive cells in the same time span (data not shown). Furthermore, a time-dependent increase in terms of number and size of mineralized nodules was observed in the PA 60, PA 67, PA 70 lines grown in OM (Figure 3B). In contrast, control cells cultured in GM did not show any calcium mineralized deposits in the same time span (data not shown).

#### 3.2.2. Adipogenic Differentiation

**Figure 4:**
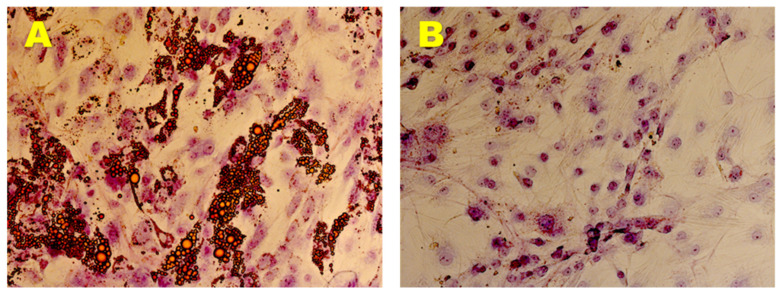
Adipogenic Differentiation Assay. Adipogenic differentiation at 35 days (**A**) and after 0 days (**B**) of induction by cytochemical staining with Oil Red O. In red the lipidic vesicles and in violet the nuclei counterstained by Toluidine Blue. Observation in brightfield. Original magnification: 20×.

Adipogenic phenotype of the established hADMSCs lines was evaluated in AM for 21 days, monitoring the formation of intracellular vesicles containing drops of lipids by Oil Red O staining. The presence of multiple intracellular lipid droplets was observed in brightfield (AxioVision, ZEISS) (Figure 4A). In contrast, control cells cultured in GM did not show any lipid vesicles in the same time span (Figure 4B).

### 3.3. Effect of 25(OH)D_3_ on the Mobilization of Intracellular Ca^2+^

**Figure 5:**
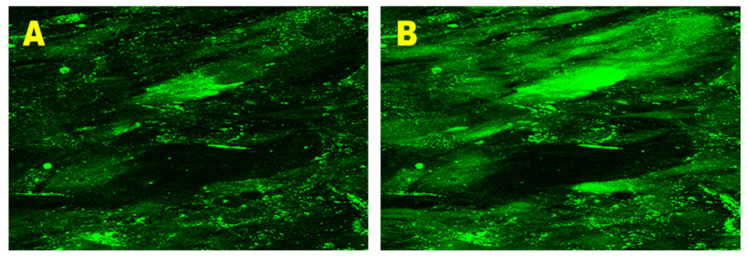
Calcium imaging on hADMSCs before (**A**) and following exposure to 10^−5^ M 25(OH)D_3_ (**B**).

**Figure 6:**
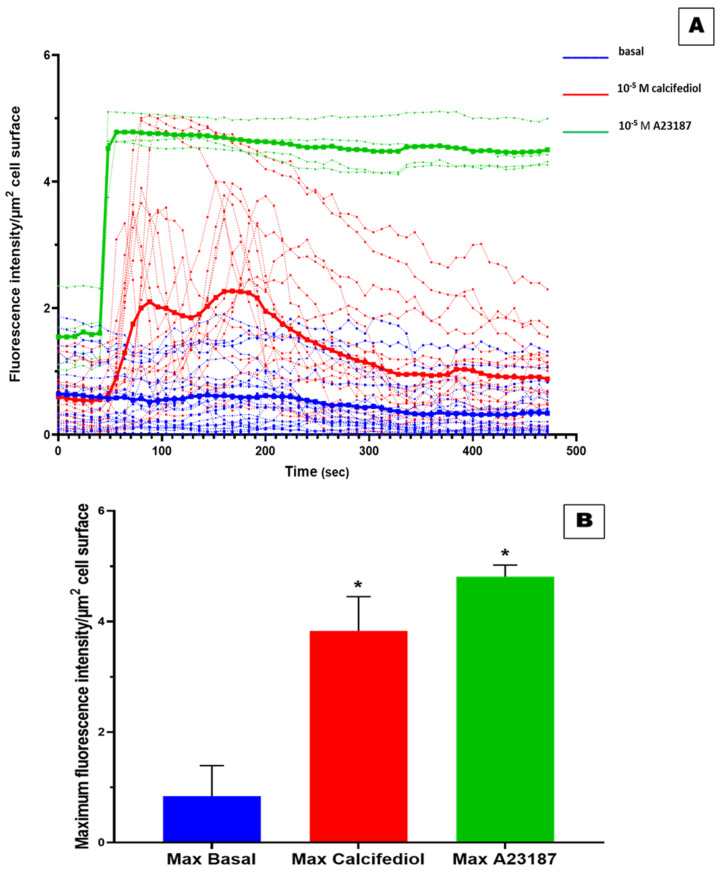
Effect of 25(OH)D_3_ on the mobilization of intracellular Ca^2+^. Time courses experiments has revealed the changes in intracellular Ca^2+^ levels in response to 25(OH)D_3_: blue for untreated cells, red for cells exposed to 10^−5^ M 25(OH)D_3_, and green for 10^−5^ M calcium ionophore-treated cells (**A**). The bold curves represent the average intensity values for Fluo-4 signals for all the cells in response to the treatment for each time (**A**). Maximum fluorescence intensity derived from cells exposed to 25(OH)D_3_ was compared to negative control (**B**). * = *p*-value < 0.0005.

To assess in vitro the capacity of 25(OH)D_3_ to trigger rapid non-genomic response, we evaluated its effects on the mobilization of intracellular Ca^2+^ levels in hADMSC by using LSCM (Figure 5). Overall, 4 cells, one for each tested cell line, responded positively to 10^−5^ M calcium ionophore A23187 as well as 17 cells treated with 10^−5^ M of 25(OH)D_3_. As shown in Figure 6, three of them reached a maximum peak comparable to that of calcium ionophore A23187 (positive control), while the other cells showed a weaker response compared to ionophore. The estimated recovery time from exposure to 10^−5^ M 25(OH)D_3_, which is the time required for the returning to the basal conditions, was about 8 min for the cells treated with 10^−5^ M 25(OH)D_3_. In contrast, in cells exposed to 10^−5^ M calcium ionophore, no recovery in the same time span was observed. The maximum peak of calcium spikes was recorded after around 48 s from the beginning of the experiment (13 s) for the 10^−5^ M calcium ionophore A23187-treated cells (5.10). Similar results were obtained in cells exposed to 10^−5^ M 25(OH)D_3_ where the maximum calcium response was reached between 64 s to 240 s (29 to 235 s from the addition of 25(OH)D_3_), varying between cells (Figure 6). The maximum fluorescence intensity derived from untreated cells was 0.84 ± 0.55 (mean ± ds), from cells exposed to 10^−5^ M of 25(OH)D_3_ was 3.83 ± 0.62 (mean ± ds), and from 10^−5^ M calcium ionophore-treated cells was 4.81 ± 0.21 (mean ± ds) (Figure 6B). The rate of increase of the maximum fluorescence intensity of the cells treated with 25(OH)D_3_ and calcium ionophore compared with cells untreated was 356% and 473%, respectively. Altogether, our experiments have revealed that the increase of intracellular Ca^2+^ levels in response to 10^−5^ M 25(OH)D_3_ was comparable to what observed with the calcium ionophore A23187 for every cell line tested.

## 4. Discussion

Vitamin D has attracted attention because its deficiency underlies the pathogenesis of well-known bone pathological conditions, such as rickets in children and osteomalacia in adults [43,44,45].

Following the identification of the molecular structure of vitamin D, a great interest has been focused on the mode of action of vitamin D at cellular level. 1α,25-(OH)_2_D_3_, the biologically active form of vitamin D, has been showed classically to mediate its effects through the interaction with a VDR, a nuclear receptor found to be expressed in virtually all cell types [1,3]. 

The discovery of non-genomic steroid actions opened to the characterization of non-genomic effects for all steroid hormones, including the sterol 1α,25-(OH)_2_D_3_ [6,7]. It is recognized that 1α,25-(OH)_2_D_3_ exerts non-genomic actions such as the activation of both intracellular signalling molecules (i.e., phospholipase A_2_ (PLA_2_), p21ras, phospholipase C, and phosphatidylinositol-3 kinase (PI3K)) and second messengers generation (i.e., cyclic AMP, phosphatidylinositol 3,4,5 trisphosphate, Ca^2+^) together with the activation of protein kinases [46,47,48,49,50]. The non-genomic mechanisms of 1α,25-(OH)_2_D_3_ encompass also the opening of Cl^−^ and Ca^2+^ channels [51].

The major difference between genomic and non-genomic actions seems to be attributable to the time to onset of action [5], with the non-genomic ones taking place within minutes, differently than genomic responses, which require the accumulation of newly formed proteins, and thus occurring in the range of hours or even days [8]. 

In addition to the rapid time course, non-genomic mechanisms are unresponsive to inhibitors of protein synthesis or transcription, such as cycloheximide and actinomycin D [5]. The identification of non-genomic effects could be made through the use of steroid bounded to large macromolecules, such as bovine serum albumin, preventing steroid molecules from entering the cell, even though the endocytosis-mediated uptake of active molecules could disprove these conclusions [8].

25(OH)D_3_, for a long time considered only a metabolic precursor of 1α,25-(OH)_2_D_3_, has been demonstrated to be an agonist VDR ligand with gene regulatory function and anti-proliferative properties despite having a reduced affinity compared with that of 1α,25-(OH)_2_D_3_ [41].

Our hypothesis has been that to test whether 25(OH)D_3_ could also activate non-genomic pathways. In particular, we analyzed in vitro the intracellular Ca^2+^ mobilization in response to 10^−5^ M 25(OH)D_3_ in MSCs derived from human adipose tissue, hADMSCs. Previous studies showed that hMSCs can differentiate into several different types of cell, such as osteoblasts, chondrocytes, adipocytes, and muscle cells [52]. Furthermore, they have been showed as good models for studying hormonal-mediated effects in vitro [42]. So, we have decided to test the non-genomic effects of 25(OH)D_3_ on previously characterized hADMSCs cell lines.

Here we show that 25(OH)D_3_ has the ability to activate rapid non-genomic pathways, such as an increase of intracellular Ca^2+^ levels, similarly to what observed with the biologically active form of vitamin D_3_ [53].

In hADMSCs loaded with Fluo-4 AM the 25(OH)D_3_ (10^−5^ M) induce a rapid (48 s) and sustained increase in intracellular Ca^2+^ concentration in line with what has been observed with the action of the secosteroid 1,25α(OH)_2_D_3_ [53].

In fact, we found that the rise of intracellular Ca^2+^ concentrations induced by 10^−5^ M 25(OH)D_3_ was 356% higher than that of untreated cells. This response was comparable to that of the calcium ionophore A23187 for every cell line tested (473% above the cells untreated).

This variation of intracellular concentration of calcium could result from an initial transient 25(OH)D_3_-induced IP3-dependent Ca^2+^ mobilization from intracellular stores into the cytoplasm followed by Ca^2+^ influx from the extracellular environment which accounts for the endorsed Ca^2+^ phase.

Regarding the 1α,25(OH)_2_D_3_, it has been proposed that the generation of non-genomic responses could be mediated by the binding of the secosteroid hormone to caveolae-associated VDR, resulting in the generation of second messengers and activation of different signal transduction pathways [54]. Recent studies have also shown that the interaction of PDIA3 and CAV1 could be involved in the activation of these rapid responses to the biologically active form of vitamin D_3_ [47,55,56]. However, the mechanisms underpinning these rapid responses to hormones at the cellular and molecular levels are not well established so far, and their elucidation could provide novel therapeutic strategies able to modulate their actions.

Another interesting non-genomic mechanism of 25(OH)D_3_ involves the processing and subsequent degradation of sterol regulatory element-binding proteins (SREBPs) cleavage-activating protein (SCAP) in the ER to control lipogenesis. Since studies provided evidence of an inverse correlation between serum levels of 25(OH)D_3_ and metabolic syndrome severity, a study of Asano et al. [57] evaluated whether SREBPs could be inhibited by secosteroids compounds. From the chemical library of substances analysed, 25(OH)D_3_ induces SCAP ubiquitin-mediated proteasomal degradation via a non-genomic mechanism independently from VDR. This mechanism results in the destabilization of SREBP thus reducing the expression of SREBP-responsive genes.

According to these findings, 25(OH)D_3_ has a non-genomic action responsible for the regulation of intracellular Ca^2+^ at concentrations that are orders of magnitude higher than those subnanomolar under normal physiological conditions. This response at a higher dose could be caused by the remarked reduced affinity of 25(OH)D_3_ approximately 500 times for VDR respect that of active form of vitamin D [58]. In this respect, subnanomolar concentrations of 25(OH)D_3_ have been demonstrated unable to trigger an increase in intracellular Ca^2+^ concentration even though a marked but delayed response was observed at higher concentrations in human spermatozoa [58].

In summary, to the best of our knowledge, this is the first report that demonstrate in vitro that 25(OH)D_3_ induce a rapid increase of intracellular Ca^2+^ levels in hADMSCs. These findings could improve the understanding of the vitamin D endocrine system, thereby paving the way for the identification of novel therapeutical targets.

## Data Availability

The data presented in this study are available on request from the corresponding author.
